# Cytotoxic impact of nicotine products on periodontal ligament cells

**DOI:** 10.1007/s00784-024-05797-x

**Published:** 2024-06-26

**Authors:** Nadine Wiesmann-Imilowski, Philipp Becker, Matthias W. Gielisch, Dirk Ziebolz, Franziska Vermehren, Marian Bitschnau, Nils Langguth, Jürgen Brieger, James Deschner, Peer W. Kämmerer

**Affiliations:** 1 Department of Oral and Maxillofacial Surgery, University Medical Center Mainz, Augustusplatz 2, 55131 Mainz, Germany; 2 Department of Otorhinolaryngology, University Medical Center Mainz, Langenbeckstrasse 1, 55131 Mainz, Germany; 3 Department of Oral and Maxillofacial Surgery, Federal Armed Forces Hospital, Ruebenacherstr. 170, 56072 Koblenz, Germany; 4 Department of Cariology, Endodontology and Periodontology, University of Leipzig, 04103 Leipzig, Germany; 5 Department of Periodontology and Operative Dentistry, University Medical Center Mainz, Augustusplatz 2, 55131 Mainz, Germany; 6 Department of Oral and Maxillofacial Surgery, Facial Plastic Surgery, University Medical Center, Johannes Gutenberg University Mainz, Augustusplatz 2, 55131 Mainz, Germany

**Keywords:** Periodontal health, Periodontal ligament cells, Electronic cigarette, Smoking, Heated tobacco product

## Abstract

**Objectives:**

The primary objective of this in vitro experiment was an assessment of proliferative capacity, metabolic activity, and potential cellular detriment of human periodontal ligament cells (hPDL) exposed to cigarette smoke (CS), electronic cigarette vapor (eCV), and heated tobacco product aerosol (HTP), or air (control).

**Materials and methods:**

Using a CAD/CAM-designed exposition chamber, hPDL were exposed to CS, eCV, HTP, or air (control) based on the Health Canada Intense Smoking Regime. Cell proliferation, metabolic activity, and cellular detriment were assessed at various time points.

**Results:**

Compared to the control, hPDL exposed to CS exhibited significantly decreased cell numbers at all time points. HTP exposure led to reduced cell numbers 48 h and 72 h post-exposure, while eCV-exposed cells showed no significant decrease. The metabolic activity of eCV-treated hPDL was slightly reduced at 7 h but recovered at 24 h and 48 h. In contrast, CS-treated cells exhibited significantly decreased metabolic activity at 24 h and 48 h, and HTP-exposed cells showed a significant decrease after 48 h. Flow cytometry indicated both apoptotic and necrotic cell death following CS exposure, with necrotic cell death being more pronounced.

**Conclusions:**

eCV and HTP demonstrated comparatively reduced detrimental effects on hPDL compared to CS.

**Clinical relevance:**

: The findings suggest that conventional cigarette smoke poses a substantial risk to periodontal health by significantly impairing cell proliferation and metabolic activity. However, alternatives such as eCV and HTP may offer a comparatively reduced risk.

## Introduction

Smoking adversely affects oral and periodontal health through various mechanisms, including compromised immune responses, impaired tissue healing, reduced mucosal blood flow, and altered bacterial colonization in the oral cavity. These factors collectively contribute to an increased risk of periodontal diseases, tooth loss, and oral cancers among smokers [1].

In conventional cigarettes, the combustion of tobacco leaves reaches temperatures ranging from 600 °C to 900 °C, resulting in the release of more than 9,500 chemicals, of which over 80 are classified as carcinogenic [2, 3]. Seeking alternatives to traditional smoking, Electronic Nicotine Delivery Systems (ENDS) have emerged as cessation aids. E-cigarettes operate by vaporizing a liquid solution known as “e-liquid” using a heating coil, producing inhalable vapor devoid of combustion by-products [4]. Due to the dissimilar composition of e-liquids compared to conventional cigarette tobacco, and the absence of combustion in e-cigarette vapor production, these devices are generally perceived as posing fewer risks to health, including periodontal health [5–10]. “Heat-not-burn” devices operate by heating tobacco to approximately 350 °C, producing a nicotine-containing aerosol without combustion. Numerous independent studies have indicated a significant reduction—up to 90%—in certain carcinogenic compounds present in heated tobacco aerosol compared to traditional cigarette smoke, while nicotine levels remain unchanged. This evidence suggests that transitioning from traditional cigarettes to heated tobacco products could potentially mitigate the risk of chronic diseases such as cardiovascular and respiratory conditions, as well as oral diseases, e.g., periodontitis or oral cancer [4, 8, 11–15].

Human periodontal ligament cells (hPDL) have shown sensitivity to chemical substances and inflammatory factors, among other exogenous stimuli [16]. Since many of the molecules contained in both conventional cigarette smoke and Electronic Nicotine Delivery Systems (ENDS) end up in the oral cavity, their levels increase in saliva relative to blood levels. They appear relevant among the molecules suspected to represent a potential threat to the regenerative potential of hPDL and possibly aggravate periodontitis [17–19]. However, to date, no studies have directly compared the effects of direct exposure of human periodontal ligament cells (hPDL) to conventional cigarette smoke, electronic cigarette vape, and the aerosol from tobacco “heat-not-burn” devices in vitro.

This in vitro study was specifically designed to address this gap in research by comprehensively examining the potential risks associated with smoking replacement products. The primary objective was to investigate the impact of cigarette smoke (CS), e-cigarette vapor (eCV), heated tobacco product (HTP) aerosol, and ambient air on key cellular parameters including cell proliferation, metabolic activity, as well as the induction of apoptosis and necrosis in hPDL. The null hypothesis was that there are no significant differences in the detrimental impact on h hPDL between CS, HTP, or eCV.

## Materials and methods

### Cell culture

Human periodontal ligament cells (hPDL) were provided by the research team led by Prof. Nicolai Miosge and isolated following established protocols as previously outlined. In brief, they were harvested from caries-free and periodontally healthy teeth, which were removed during wisdom tooth surgery or which had to be extracted for orthodontic reasons [20]. Cultivation was conducted using a standard medium composed of Dulbecco’s Modified Eagle’s Medium supplemented with 10% fetal calf serum, 1% Penicillin/Streptomycin, and 1% L-glutamine (Gibco Invitrogen, Darmstadt, Germany). The cells were maintained at 37 °C under conditions of 95% humidity and 5% CO2. Subsequent to reaching 80–90% confluence, hPDL passaging was executed using Accutase Enzyme Cell Detachment Medium (Sigma-Aldrich, St. Louis, MO, US) until the seventh passage. Following cell counting, they were allocated into four subgroups (eCV, CS, HTP, ambient air (control group)), and seeded into 6-well plates at a density of 50,000 cells per well.

### Exposure chambers and process of smoke and aerosol exposure

**Fig. 1:**
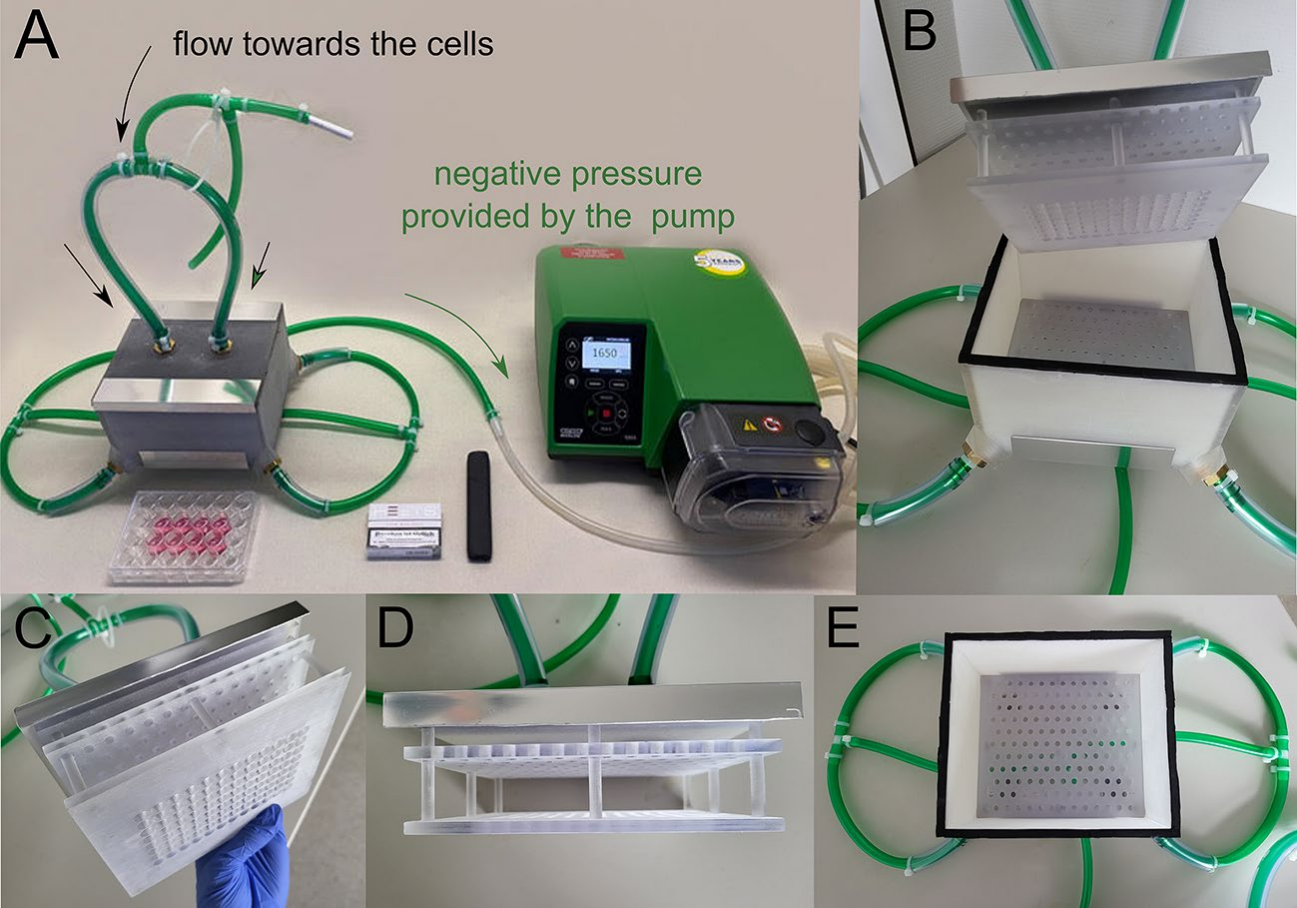
Experimental setup. **A** On the left, the exposition chamber with an attached cigarette is shown. It is connected via a tubing system with a three-way valve to a peristaltic pump (on the right), which generates negative pressure to produce equal puffs. **B** The aerosol flows into the exposition chamber through two inlets and is evenly distributed to the cells at the top of the chamber by a two-stage system of smoke diffusers. **C and D** Two-stage system of smoke diffusers. **E** At the bottom of the chamber, the aerosol is also channeled out evenly through a diffuser and discharged at all four corners

To facilitate the exposure of cells to smoke and aerosols, custom-designed computer-aided manufactured (CAD/CAM) exposure chambers were created in-house using FreeCAD software (Version 0.20.1) and manufactured with MED610 material (Stratasys, Minnesota, United States) via Objet Eden 260 3D-printer (Stratasys, MN, US). Each chamber features four outlets, converging into a single tube connected to a Watson-Marlow 530 S vacuum pump (Watson-Marlow, Rommerskirchen, Germany) (Fig. 1). Smoke diffusers were integrated into each chamber to ensure uniform distribution of smoke and aerosols among the cells. The smoking device was linked to an individual adapter connecting the chamber, with a closable outlet tube for suction. The pump’s volume flow rate was fixed at 1,650 ml/min [21]. hPDL cells prepared in 6-well plates were positioned within the chamber at predetermined locations, guided by markers on the chamber base to ensure reproducibility.

Cigarette smoke (CS) was generated using Marlboro Red cigarettes (Philip Morris, Munich, Germany). An SMOK G-PRIV 2 Mod 230 W smoking device (Smok, Smoktech, Shenzhen IVPS Technology Co. Ltd., Shenzhen, China) operating at 30 W in conjunction with a Melo 2 clearomizer and a Ni 0.15-ohm coil produced e-cigarette vapor (eCV). The e-cigarette liquid composition included a base consisting of 55% propylene glycol E1520, 35% glycerin E433, and 10% H2O (Advanced Base Liquid), combined with 3 mg/ml nicotine, along with passion fruit/peach liquid (both Riccardo Retail, Neubrandenburg, Germany). For the generation of heated tobacco product aerosol (HTP), HEETS were utilized in combination with an IQOS 3 Multi tobacco heating system (both Philip Morris International, New York) [11, 22]. The Health Canada Intense Smoking Regime [23] was strictly adhered to for cell exposure to the different aerosols. This regime specifies a puff volume of 55 ml over 2 s for each puff, with twelve puffs executed over 6 min, corresponding to one puff every 30 s. A timer regulated each puff by closing the three-way valve connecting the device, smoking chamber, and air supply for 2 s, ensuring consistent exposure to CS, eCV, or HTP. Following exposure, samples were stored separately in incubators set at 37 °C and 5% CO2 to prevent contamination, mutual interference, or distortion of results.

### Cell proliferation

The hPDL were stained with a fluorescent dye (CellTracker™ Red CMTPX dye, Invitrogen, Darmstadt, Germany; catalog number: C34552) at 24 h, 48 h, and 72 h post-exposure to CS, eCV, HTP, or ambient air. Of importance is that an assessment of the early proliferative response following exposure can be taken immediately after 24 h. This gives a clear idea of the instant effect of the exposure on cell division and early cellular behavior changes. The time point at 48 h is significant in comprehending whether the initially observed changes at 24 h will be sustained, grow, or decline with time. The later time point of 72 h is important to assess the sustained impact of the exposure to cell proliferation. It helps determine longer-term trends and potential cumulative effects, giving a more complete picture of the cellular response over several cell cycles.

Following the manufacturer’s staining protocol, the supernatant was aspirated, and 3.6 µl of dye solution was added to 1 ml of serum-free culture medium in each well. The solution was replaced with a fresh medium after a 30-minute incubation period at 37 °C and 5% CO2. Subsequently, four predetermined areas within each well were selected and examined using a fluorescence microscope (Biorevo BZ-9000, Keyence, Neu Isenburg, Germany) at 10x magnification, utilizing the corresponding software (BZ-II Viewer Biorevo BZ-9000, Keyence). Cell quantification was performed using ImageJ software (NIH, Bethesda, MD, USA).

### Cellular metabolic activity

A resazurin-based colorimetric agent (AlamarBlue™ Cell Viability Reagent, Invitrogen, Darmstadt, Germany; catalog number: A50100 and A50101) was employed at 7 h, 24 h, and 48 h time points, following the manufacturer’s instructions. Here, the 7 h, 24 h, and 48 h time points were selected based on several combined factors that captured immediate and longer-term cellular responses to exposure. More precisely, the 7 h early time point was determined to measure the immediate reaction of cells to exposure in terms of initial changes in metabolism and early markers of cell stress or damage. This is because, at 24 h, continuing cellular metabolic effects of the exposure would be considered measurable. So, this time point would help verify whether metabolic changes that appear at 7 h are a transient response or remain permanent. Using the later time point of 48 h, we tried to give a complete overview of dynamic changes in cellular activities with metabolism, starting from immediate reactions to more sustained effects.

Briefly, the cell culture medium was replaced with medium containing 10% AlamarBlue™ 5 h before each measurement, and cells were subsequently incubated at 37 °C and 5% CO2. Emission was then measured at a wavelength of 600 nm with excitation at 538 nm using a Multi-Mode Microplate Reader (SpectraMax iD5, Molecular Devices, San Jose, CA, US) along with the corresponding software (SoftMax Pro Version 7, Molecular Devices, San Jose, CA, US).

### Flow cytometric (FACS) assessment of apoptosis and necrosis

After cellular exposure, followed by a 24-hour incubation period at 37 °C and 5% CO2 in the incubator, hPDL were detached using Accutase Enzyme Cell Detachment Medium and stained with propidium iodide (PI) (Thermo Fisher Scientific, Waltham, MA, US) and Annexin V-APC (Thermo Fisher Scientific, Waltham, MA, US) according to the manufacturer’s protocol. Subsequently, 10,000 cells per sample were analyzed using a BD FACS Canto II flow cytometer (Becton Dickinson, Franklin Lakes, NY, US). Annexin V-APC served as a fluorescence marker for the exposure of phosphatidylserine, located on the outer cellular membrane during apoptotic processes. PI was utilized as a fluorescence marker for the integrity of the outer cellular membrane, indicative of necrosis or late cell death. Cells lacking staining were categorized as alive, those stained solely by PI as necrotic, stained exclusively by Annexin V-APC as apoptotic, and stained by both PI and Annexin V-APC as dead. Two positive controls were incorporated into the measurement: one for apoptosis (incubation with 10 µM staurosporine for 14 h) and one for necrosis (incubation with 2,400 µM H2O2 for 2 h) to validate the correct staining of the cell panel. Additionally, corresponding unstained controls and single stains were conducted to correct for autofluorescent effects post-treatment with the indicated aerosols. The acquired data were analyzed using the cytobank platform (https://www.cy-tobank.org/, Cytobank, Inc., Santa Clara, CA, US).

### Statistical analysis

Each experiment was conducted in a minimum of three independent runs. Data were evaluated for minimum, maximum, median, mean values, skewness, quartiles, and standard deviations. Illustrations were conducted using boxplots and bar charts. In further exploratory data analysis, the Kolmogorov-Smirnov test assessed differences between groups. The Mann-Whitney-U test was employed for p-values less than 0.05, whereas for p-values greater than 0.05, a Student’s t-test for independent samples was utilized. A multivariable analysis was conducted for confirmatory evaluation, incorporating Bonferroni correction for multiple testing. A p-value below 0.05 was considered statistically significant. All statistical analyses and graphical representations were carried out using GraphPad Prism, Version 6 (GraphPad Software, Inc., San Diego, CA, US).

## Results

### Cell proliferation

**Fig. 2:**
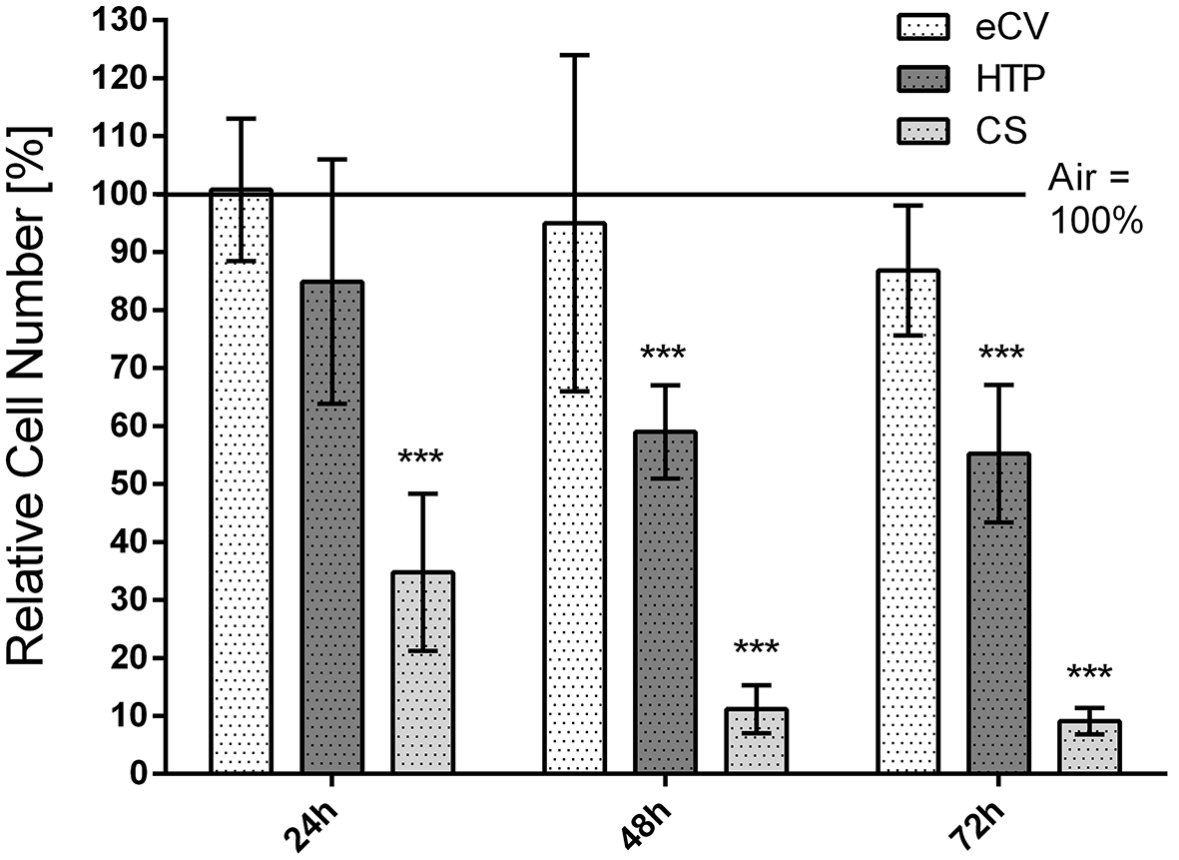
hPDL were stained and counted 24 h, 48 h, and 72 h after treatment with eCV, HTP, CS, or ambient air. Shown are mean values and standard deviations. The control (ambient air) was set to 100% at each point in time, and the test groups are shown in relation to this control. (*=*p* < 0,05; **=*p* < 0,01; ***=*p* < 0,001)

Human periodontal ligament cells (hPDL) were stained at 24 h, 48 h, and 72 h post-exposure to cigarette smoke (CS), e-cigarette vapor (eCV), heated tobacco product (HTP), or ambient air using CellTracker Red to assess cell proliferation (Fig. 2). Cells exposed to CS exhibited significantly lower cell numbers, indicating reduced proliferation, compared to control cells at all investigated time points (24 h, 48 h, and 72 h: *p* < 0.001). Similarly, HTP-treated cells demonstrated significantly reduced cell numbers after 48 h (*p* < 0.001) and 72 h (*p* < 0.001) compared to control cells. In contrast, there was no significant reduction in cell numbers observed in eCV-treated cells compared to cells exposed to ambient air.

### Cellular metabolic activity

**Fig. 3:**
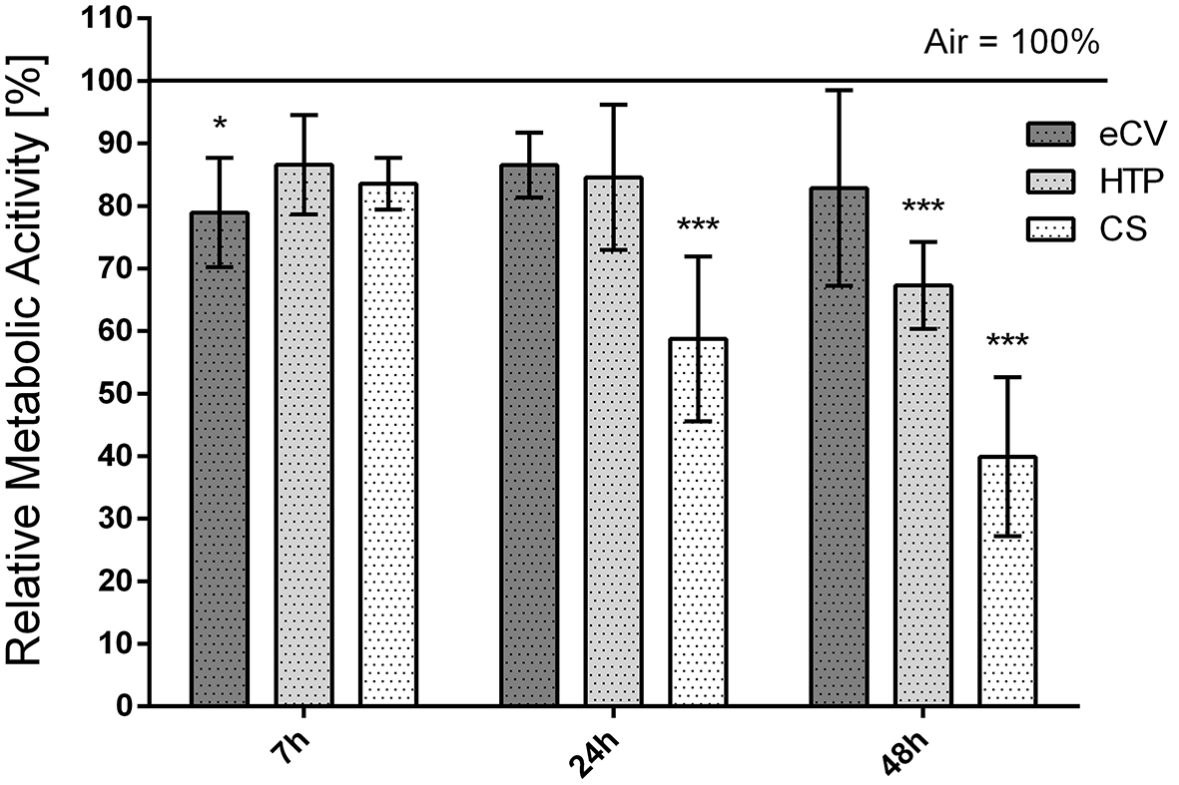
To assess the cellular metabolic activity after exposure to eCV, HTP, CS, or ambient air, cells were stained with the resazurin-based AlamarBlue™ assay after 7 h, 24 h, and 48 h. Shown are mean values and standard deviations. The control (ambient air) was set to 100% at each point in time, and the test groups are shown in relation to this control. (*=*p* < 0,05; **=*p* < 0,01; ***=*p* < 0,001)

To evaluate cellular metabolic activity, hPDL were analyzed using the resazurin-based AlamarBlue™ assay at 7 h, 24 h, and 48 h post-exposure to CS, eCV, HTP, or ambient air (Fig. 3). Cells exposed to cigarette smoke exhibited significantly decreased metabolic activity after 24 h and 48 h compared to control (*p* < 0.001), indicating a substantial loss in cellular viability. HTP-treated cells also displayed a notable decline in cellular metabolic activity after 48 h (*p* < 0.001), consistent with the cell count findings. Conversely, cells exposed to eCV demonstrated an initial decrease in cellular viability after 7 h (*p* = 0.023). However, no significant difference was observed at 24 h and 48 h post-exposure compared to control cells.

### Apoptosis and necrosis

**Fig. 4:**
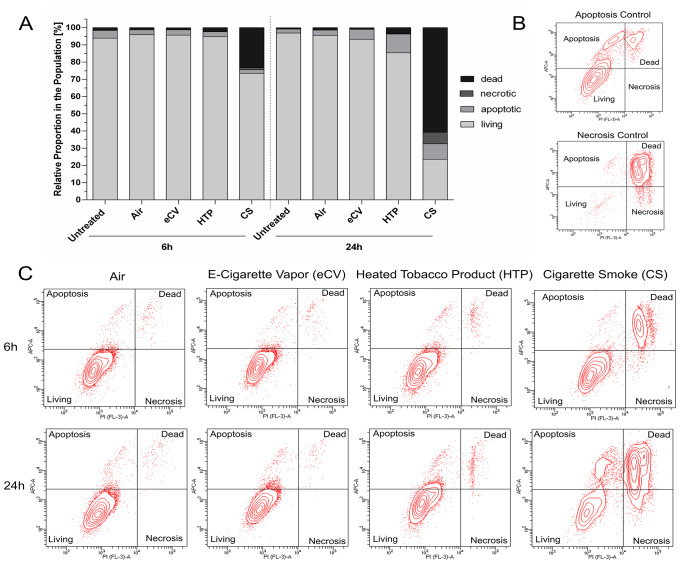
Flow cytometry was used to assess and understand the mechanism of cell death following exposure to the different aerosols. **A** shows the ratios between living, dead, apoptotic, and necrotic cells in the cell population as columns. Shown are mean values, while standard deviations were not plotted in favor of clarity. **B** shows a panel of stained cells of the two cell death controls, and **C** represents the stained cells of the treatment groups under investigation. No significant differences between the groups were seen

Flow cytometry (FACS) was utilized to evaluate apoptosis and necrosis of hPDL exposed to CS, eCV, HTP, or ambient air, analyzed at 24 h post-exposure via staining with propidium iodide for cellular integrity and AnnexinV-APC to assess apoptotic processes. Figure 4B and C depict a representative panel of stained cells treated with the indicated aerosols. The intact, living cells are situated in the lower left quadrant, while the (early) apoptotic cells are located in the upper left quadrant, dead cells (either late apoptotic or necrotic) are positioned in the upper right quadrant, and necrotic cells reside in the lower right quadrant. Two positive controls, one for apoptosis (staurosporine) and one for necrosis (H2O2), were included in the measurement to ensure correct staining of the cell panel (Fig. 4B). Although not statistically significant, there was an observable decrease in living cells following exposure to CS (Fig. 4A), along with a notable increase in dead cells and a slight elevation in apoptotic and necrotic cell death. In contrast, minimal to no shift in the ratio between different cell populations was observed in cells treated with eCV and HTP.

## Discussion

The effects of cigarette smoke (CS) compared to various commercially available tobacco substitutes on human periodontal ligament cells (hPDL) remain unexplored. Thus, the aim of this study was to investigate how exposures to electronic cigarette vapor (eCV) and heated tobacco product (HTP), in comparison to CS and air, impact hPDL in terms of their proliferation, metabolic activity, and cell death.

Periodontal ligament cells play a crucial role in periodontal health by producing collagen and contributing to forming and regenerating periodontal connective tissue. They also function as essential immune system components, releasing cytokines in response to pathogenic exposure, such as those found in CS or eCV [17–19]. The metabolic function of hPDL can significantly impact the success of systematic periodontal therapy [24, 25].

In this study, we observed a notable reduction in the metabolic activity of hPDL cells exposed to CS within 24 h, with levels dropping to less than 50% compared to the air-exposed control by the 48-hour mark. These findings underscore the sensitivity of hPDL to CS and suggest that CS exposure could significantly impede periodontal regeneration. Similarly, the metabolic activity of cells exposed to HTP was significantly diminished after 48 h compared to the control group, although no significant differences were observed at earlier time points. This indicates that the impact of HTP on hPDL may be less severe than that of CS. Conversely, in the group exposed to eCV, we noted a decrease in metabolic activity after 7 h, with no significant changes observed at 24 and 48 h. Considering the in vitro nature of this study, these results align with existing findings suggesting that eCV may primarily contribute to acute damage, such as acute nicotine poisoning, rather than causing prolonged effects [6, 26]. A systematic review indicated that nicotine concentrations encountered in in vitro studies generally lack cytotoxicity on hPDL, suggesting that nicotine may not be the primary factor responsible for cellular damage [27]. In contrast, the levels of nicotine found in the saliva of users of smokeless tobacco products have been shown to reach cytotoxic thresholds [28]. Despite this, the injurious consequences of conventional tobacco smoking remain the most severe. For patients with periodontitis who continue to smoke and are unable to quit, the use of eCV or HTP may present a relatively milder alternative to conventional CS.

Cell proliferation was significantly reduced in the CS-treated cells at all measurement times, massively after 48 h and even to less than 10% after 72 h compared to the control group. After 48 h and 72 h, the HTP group showed significantly reduced cell proliferation compared to the control group but remained at an average of over 50% even after 72 h. In the eCV group, the relative cell number was not reduced at all. A comparable study by Pagano et al. demonstrated a proliferation stimulation of oral fibroblasts and keratinocytes by HTP [29]. In one of our previous studies, a metabolic stimulation of human gingival fibroblasts by eCV was also detectable [21]. In the context of interpreting the findings from this study, it is conceivable that reduced metabolic activity may impede the regenerative capacity of the periodontal tissues. Nevertheless, it is essential to acknowledge that an elevated proliferation rate, albeit only occasionally observed in this study and without statistical significance, is recognized as a (soft) criterion for malignancy. This aspect warrants further investigation in future research endeavors. It may be imperative to ascertain whether specific components present in HTP and eCV have the potential to induce an elevated proliferation rate.

To realistically simulate the process of smoking and inhalation in vitro, a whole smoke exposure chamber was first developed for use in early tests. CS, eCV, HTP, and air were introduced into the square-bottomed chamber via two tubes attached directly above the wells. The standardized protocol was additionally supported by the strict use of The Health Canada Intense Smoking Regim [23]. A counterargument to this model could be that hPDL in the human body are not covered with a liquid medium as in cell cultures. On the other hand, PDL cells, at least in healthy human individuals, are usually not directly exposed to harmful substances but are still covered by sulcus fluid, which in turn comes very close to the simulation through the exposition chamber. Another established method consists of first preparing a smoke extract. Here, smoke is passed through a filter, and the filtered substances are converted into a liquid, which is then added to the cell culture [30]. The major disadvantage of this conventional method is that the cells to be examined are only exposed to the particle phase, not the vapor phase of the smoke. However, the smoke extract is not representative of the entire smoke aerosol. In contrast, in whole smoke exposure systems, where the particulate and vapor phases are considered, the smoke’s physiological composition is considered [30–32]. St-Laurent et al. showed in a standardized in vitro design that the choice of experimental setup (cigarette smoke extract versus smoking chamber) has a decisive influence on the test results [33]. The presence of diverse and heterogeneous experimental designs poses challenges when comparing the outcomes of various smoking studies. Particularly within e-cigarettes and their vapor, the wide range of available vaporizers, each offering adjustable settings, and the multitude of e-liquids on the market contribute to the complexity and variability in research protocols.

In summary, the findings from this in vitro study provide evidence to reject, at least partially, the null hypothesis, asserting no significant differences in the detrimental impact on hPDL between CS, HTP, or eCV. The most pronounced proliferation-inhibiting and cytotoxic effects were observed in response to exposure to conventional cigarette smoke. Conversely, tobacco-free eCV exhibited the least pronounced impact on hPDL in vitro. The adverse effects of heated tobacco product aerosol fell intermediate between the two. It is essential to note that, in contrast to e-cigarettes, heated tobacco products are subject to standardization and legal regulation, and users must adhere to specified technical settings.

In the clinical context, this in vitro study’s findings align with and provide mechanistic insights that could elucidate various clinical observations in periodontal health. Firstly, our results corroborate findings from clinical studies indicating that smokers exhibit poorer responses to periodontal therapy compared to non-smokers [34]. Secondly, the direct positive impact of smoking cessation on periodontal conditions following therapy, as observed in former smokers compared to non-smokers, is supported by our data [34]. Additionally, our study adds nuance to the understanding of electronic cigarette use, as evidenced by comparable clinical periodontal findings between e-cigarette smokers and non-smokers [9, 10]. Furthermore, the hierarchy of clinical outcomes following periodontal therapy—wherein non-smokers demonstrate superior or comparable results to e-cigarette smokers, who in turn fare better than smokers—is substantiated by our findings [9, 10, 35, 36].

## Conclusion

The study thus underlines the clinical importance of understanding the differential impact of CS, eCV, and HTP aerosols on hPDL cells. Periodontal ligament cells are in the front line while maintaining periodontal health and tissue regeneration. Data suggests relative risks in periodontal health: CS causes substantial impairment of cell proliferation and metabolic activity; relative risks from alternatives are likely to be less. These data would be significant for dental clinicians and public health decision-makers, who can warn people about the prospective risks accompanying smoking products. Additionally, awareness of these different impacts can help develop targeted therapies and preventive strategies for smokers, which may improve periodontal treatment and oral health outcomes.

## Data Availability

Data are available upon request from the corresponding author.

## References

[1] Zhang Y, He J, He B, Huang R, Li M (2019) Effect of tobacco on periodontal disease and oral cancer. Tob Induc Dis 17:40. 10.18332/tid/106187 31516483 10.18332/tid/106187 PMC6662776

[2] Li Y, Hecht SS (2022) Carcinogenic components of tobacco and tobacco smoke: a 2022 update. Food Chem Toxicol 165:113179. 10.1016/j.fct.2022.113179 35643228 10.1016/j.fct.2022.113179 PMC9616535

[3] Vukas J, Mallock-Ohnesorg N, Ruther T, Pieper E, Romano-Brandt L, Stoll Y, Hoehne L, Burgmann N, Laux P, Luch A, Rabenstein A (2023) Two different heated Tobacco products vs. cigarettes: comparison of Nicotine Delivery and Subjective effects in experienced users. Toxics 11. 10.3390/toxics11060525 10.3390/toxics11060525 PMC10301154 37368625

[4] Ebersole J, Samburova V, Son Y, Cappelli D, Demopoulos C, Capurro A, Pinto A, Chrzan B, Kingsley K, Howard K, Clark N, Khlystov A (2020) Harmful chemicals emitted from electronic cigarettes and potential deleterious effects in the oral cavity. Tob Induc Dis 18:41. 10.18332/tid/116988 32435175 10.18332/tid/116988 PMC7233525

[5] Harrell PT, Marquinez NS, Correa JB, Meltzer LR, Unrod M, Sutton SK, Simmons VN, Brandon TH (2015) Expectancies for cigarettes, e-cigarettes, and nicotine replacement therapies among e-cigarette users (aka vapers). Nicotine Tob Res 17:193–200. 10.1093/ntr/ntu149 25168035 10.1093/ntr/ntu149 PMC4438353

[6] Schraufnagel DE, Blasi F, Drummond MB, Lam DC, Latif E, Rosen MJ, Sansores R, Van Zyl-Smit R (2014) Electronic cigarettes. A position statement of the forum of international respiratory societies. Am J Respir Crit Care Med 190:611–618. 10.1164/rccm.201407-1198PP 25006874 10.1164/rccm.201407-1198PP

[7] Thiem DGE, Donkiewicz P, Rejaey R, Wiesmann-Imilowski N, Deschner J, Al-Nawas B, Kämmerer PW (2023) The impact of electronic and conventional cigarettes on periodontal health-a systematic review and meta-analysis. Clin Oral Investig 27:4911–4928. 10.1007/s00784-023-05162-4 37526741 10.1007/s00784-023-05162-4 PMC10492702

[8] Holliday R, Chaffee BW, Jakubovics NS, Kist R, Preshaw PM (2021) Electronic cigarettes and oral health. J Dent Res 100:906–913. 10.1177/00220345211002116 33764176 10.1177/00220345211002116 PMC8293737

[9] Pesce P, Menini M, Ugo G, Bagnasco F, Dioguardi M, Troiano G (2022) Evaluation of periodontal indices among non-smokers, tobacco, and e-cigarette smokers: a systematic review and network meta-analysis. Clin Oral Investig 26:4701–4714. 10.1007/s00784-022-04531-9 35556173 10.1007/s00784-022-04531-9 PMC9276554

[10] Robbins J, Ali K (2022) How do periodontal indices compare among non-smokers, tobacco and e-cigarette smokers? Evid Based Dent 23:116–117. 10.1038/s41432-022-0809-y 36151288 10.1038/s41432-022-0809-y

[11] Davis B, Williams M, Talbot P (2019) iQOS: evidence of pyrolysis and release of a toxicant from plastic. Tob Control 28:34–41. 10.1136/tobaccocontrol-2017-054104 29535257 10.1136/tobaccocontrol-2017-054104

[12] Farsalinos KE, Yannovits N, Sarri T, Voudris V, Poulas K, Leischow SJ (2018) Carbonyl emissions from a novel heated tobacco product (IQOS): comparison with an e-cigarette and a tobacco cigarette. Addiction 113:2099–2106. 10.1111/add.14365 29920842 10.1111/add.14365

[13] Li X, Luo Y, Jiang X, Zhang H, Zhu F, Hu S, Hou H, Hu Q, Pang Y (2019) Chemical Analysis and simulated pyrolysis of Tobacco Heating System 2.2 compared to conventional cigarettes. Nicotine Tob Res 21:111–118. 10.1093/ntr/nty005 29319815 10.1093/ntr/nty005

[14] Mallock N, Boss L, Burk R, Danziger M, Welsch T, Hahn H, Trieu HL, Hahn J, Pieper E, Henkler-Stephani F, Hutzler C, Luch A (2018) Levels of selected analytes in the emissions of heat not burn tobacco products that are relevant to assess human health risks. Arch Toxicol 92:2145–2149. 10.1007/s00204-018-2215-y 29730817 10.1007/s00204-018-2215-y PMC6002459

[15] Znyk M, Jurewicz J, Kaleta D (2021) Exposure to Heated Tobacco Products and Adverse Health Effects, a systematic review. Int J Environ Res Public Health 18. 10.3390/ijerph18126651 10.3390/ijerph18126651 PMC8296358 34205612

[16] Lallier TE, Moylan JT, Maturin E (2017) Greater sensitivity of oral fibroblasts to smoked Versus Smokeless Tobacco. J Periodontol 88:1356–1365. 10.1902/jop.2017.170232 28708037 10.1902/jop.2017.170232

[17] Jiang Y, Yang K, Jia B, Gao Y, Chen Y, Chen P, Lu X, Zhang W, Wang X (2024) Nicotine destructs dental stem cell-based periodontal tissue regeneration. J Dent Sci 19:231–245. 10.1016/j.jds.2023.04.018 38303843 10.1016/j.jds.2023.04.018 PMC10829564

[18] Shen Y, Liu C, Yang T, Tang Y, Shen Y, Gu Y (2023) Transcriptome characterization of human gingival mesenchymal and periodontal ligament stem cells in response to electronic-cigarettes. Environ Pollut 323:121307. 10.1016/j.envpol.2023.121307 36804562 10.1016/j.envpol.2023.121307

[19] Mourao CF, Shibli JA (2023) What is the impact of e-cigarettes on periodontal stem cells as revealed by transcriptomic analyses? Evid Based Dent 24:168–169. 10.1038/s41432-023-00939-8 37814004 10.1038/s41432-023-00939-8

[20] Rath-Deschner B, Nogueira AVB, Memmert S, Nokhbehsaim M, Augusto Cirelli J, Eick S, Miosge N, Kirschneck C, Kesting M, Deschner J, Jager A, Damanaki A (2021) Regulation of anti-apoptotic SOD2 and BIRC3 in Periodontal cells and tissues. Int J Mol Sci 22. 10.3390/ijms22020591 10.3390/ijms22020591 PMC7827060 33435582

[21] Vermehren MF, Wiesmann N, Deschner J, Brieger J, Al-Nawas B, Kämmerer PW (2020) Comparative analysis of the impact of e-cigarette vapor and cigarette smoke on human gingival fibroblasts. Toxicol Vitro 69:105005. 10.1016/j.tiv.2020.105005 10.1016/j.tiv.2020.105005 32956835

[22] Smith MR, Clark B, Ludicke F, Schaller JP, Vanscheeuwijck P, Hoeng J, Peitsch MC (2016) Evaluation of the Tobacco Heating System 2.2. Part 1: description of the system and the scientific assessment program. Regul Toxicol Pharmacol 81(Suppl 2):S17–S26. 10.1016/j.yrtph.2016.07.006 27450400 10.1016/j.yrtph.2016.07.006

[23] Belushkin M, Esposito M, Jaccard G, Jeannet C, Korneliou A, Tafin Djoko D (2018) Role of testing standards in smoke-free product assessments. Regul Toxicol Pharmacol 98:1–8. 10.1016/j.yrtph.2018.06.021 29983383 10.1016/j.yrtph.2018.06.021

[24] Jonsson D, Nebel D, Bratthall G, Nilsson BO (2011) The human periodontal ligament cell: a fibroblast-like cell acting as an immune cell. J Periodontal Res 46:153–157. 10.1111/j.1600-0765.2010.01331.x 21118418 10.1111/j.1600-0765.2010.01331.x

[25] Lin Y, Tang Z, Jin L, Yang Y (2022) The expression and Regulatory roles of Long non-coding RNAs in Periodontal Ligament cells: a systematic review. Biomolecules 12:304 35204802 10.3390/biom12020304 PMC8869287

[26] Rom O, Pecorelli A, Valacchi G, Reznick AZ (2015) Are E-cigarettes a safe and good alternative to cigarette smoking? Ann N Y Acad Sci 1340:65–74. 10.1111/nyas.12609 25557889 10.1111/nyas.12609

[27] Holliday RS, Campbell J, Preshaw PM (2019) Effect of nicotine on human gingival, periodontal ligament and oral epithelial cells. A systematic review of the literature. J Dent 86:81–88. 10.1016/j.jdent.2019.05.030 31136818 10.1016/j.jdent.2019.05.030

[28] Moore D (2020) Is nicotine damaging to oral tissues? Evid Based Dent 21:32–33. 10.1038/s41432-020-0082-x 32221495 10.1038/s41432-020-0082-x

[29] Pagano S, Negri P, Coniglio M, Bruscoli S, Di Michele A, Marchetti MC, Valenti C, Gambelunghe A, Fanasca L, Billi M, Cianetti S, Marinucci L (2021) Heat-not-burn tobacco (IQOS), oral fibroblasts and keratinocytes: cytotoxicity, morphological analysis, apoptosis and cellular cycle. An in vitro study. J Periodontal Res 56:917–928. 10.1111/jre.12888 34018192 10.1111/jre.12888 PMC8518503

[30] Crooks I, Dillon DM, Scott JK, Ballantyne M, Meredith C (2013) The effect of long term storage on tobacco smoke particulate matter in in vitro genotoxicity and cytotoxicity assays. Regul Toxicol Pharmacol 65:196–200. 10.1016/j.yrtph.2012.11.012 23220485 10.1016/j.yrtph.2012.11.012

[31] Fukano Y, Ogura M, Eguchi K, Shibagaki M, Suzuki M (2004) Modified procedure of a direct in vitro exposure system for mammalian cells to whole cigarette smoke. Exp Toxicol Pathol 55:317–323. 10.1078/0940-2993-00341 15088633 10.1078/0940-2993-00341

[32] Thorne D, Adamson J (2013) A review of in vitro cigarette smoke exposure systems. Exp Toxicol Pathol 65:1183–1193. 10.1016/j.etp.2013.06.001 23850067 10.1016/j.etp.2013.06.001

[33] St-Laurent J, Proulx LI, Boulet LP, Bissonnette E (2009) Comparison of two in vitro models of cigarette smoke exposure. Inhal Toxicol 21:1148–1153. 10.3109/08958370902926692 19852558 10.3109/08958370902926692

[34] Leite FRM, Nascimento GG, Baake S, Pedersen LD, Scheutz F, Lopez R (2019) Impact of Smoking Cessation on Periodontitis: a systematic review and Meta-analysis of prospective longitudinal observational and interventional studies. Nicotine Tob Res 21:1600–1608. 10.1093/ntr/nty147 30011036 10.1093/ntr/nty147

[35] ALHarthi SS, BinShabaib M, Akram Z, Rahman I, Romanos GE, Javed F (2019) Impact of cigarette smoking and vaping on the outcome of full-mouth ultrasonic scaling among patients with gingival inflammation: a prospective study. Clin Oral Investig 23:2751–2758. 10.1007/s00784-018-2725-2 30361795 10.1007/s00784-018-2725-2

[36] Al-Hamoudi N, Alsahhaf A, Al Deeb M, Alrabiah M, Vohra F, Abduljabbar T (2020) Effect of scaling and root planing on the expression of anti-inflammatory cytokines (IL-4, IL-9, IL-10, and IL-13) in the gingival crevicular fluid of electronic cigarette users and non-smokers with moderate chronic periodontitis. J Periodontal Implant Sci 50:74–82. 10.5051/jpis.2020.50.2.74 32395386 10.5051/jpis.2020.50.2.74 PMC7192822

